# A New Crank Arm-Based Load Cell for the 3D Analysis of the Force Applied by a Cyclist

**DOI:** 10.3390/s141222921

**Published:** 2014-12-03

**Authors:** Alexandre Balbinot, Cleiton Milani, Jussan da Silva Bahia Nascimento

**Affiliations:** Electrical Engineering, Instrumentation Laboratory, Federal University of Rio Grande do Sul, Avenue Osvaldo Aranha 103, Porto Alegre 90035-190, Brazil; E-Mails: cleiton_milani@hotmail.com (C.M.); jussan.sbn@gmail.com (J.S.B.N.)

**Keywords:** biomechanics instrumentation, load cell, cycling, Bluetooth

## Abstract

This report describes a new crank arm-based force platform designed to evaluate the three-dimensional force applied to the pedals by cyclists in real conditions. The force platform was designed to be fitted on a conventional competition bicycle crankset while data is transmitted wirelessly through a Bluetooth^TM^ module and also stored on a SD card. A 3D solid model is created in the SolidWorks (Dassault Systèmes SOLIDWORKS Corp.) to analyze the static and dynamic characteristics of the crank arm by using the finite elements technique. Each crankset arm is used as a load cell based on strain gauges configured as three Wheatstone bridges. The signals are conditioned on a printed circuit board attached directly to the structure. The load cell showed a maximum nonlinearity error between 0.36% and 0.61% and a maximum uncertainty of 2.3% referred to the sensitivity of each channel. A roller trainer equipped with an optical encoder was also developed, allowing the measurement of the wheel's instantaneous velocity.

## 1. Introduction

In cycling, the system formed by the cyclist and the bicycle presents several technical characteristics that may influence elite athletes' performance. This opens the possibility of applying and studying biomedical instrumentation related to the pedaling motion. Among these characteristics, the studies of cadence [1], forces applied to the pedal [2], and positioning of the saddle and handlebars [3] can be highlighted. Biomechanical studies of motion and applied forces are usually conducted in laboratory with the aid of training platforms such as cycle ergometers or roller trainers in order to facilitate the reproducibility of test conditions. With information such as the force applied to the pedal and, thus, the generated mechanical power, professional cycling teams can validate training techniques and evaluate the influence of variables such as the cyclist's stance, the pedaling cadence, the asymmetry between forces, and other parameters of interest. Although the industry already offers equipment capable of measuring the athlete's resultant produced power, there are no models capable of providing information on the components of the applied force, so their development is restricted to research laboratories [4,5]. Therefore, the main goal of this study is the development of a force platform that allows the analysis of the three-dimensional components of the force applied to the crank by the cyclist during real competition conditions. A secondary goal was the development of an instrumented roller trainer capable of measuring the wheel's instantaneous velocity during laboratory trials.

## 2. Basic Mechanical Concepts

**Figure 1.**
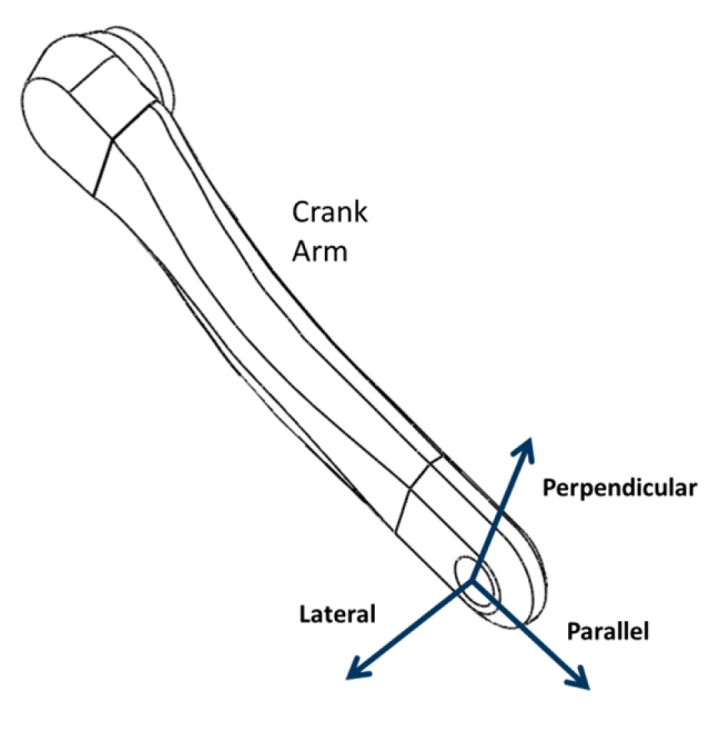
Components of the applied force.

By applying a force to the pedal, the cyclist generates a rotating motion of the crank arm around the crankset axle. The biomechanical studies about this applied force became popular after the studies of Hull *et al.* [6–8], when an instrumented pedal capable of measuring the force components in 3D was proposed, enabling the development of more advanced kinetic models. The force applied to the pedal is transferred to the crank arm, and it can be decomposed into three main components, as shown in Figure 1:

‐ Perpendicular to the crank arm, acting in the rotation plane. This is the only effective component, *i.e.*, able to rotate the crank arm [9];
‐ Parallel to the crank arm;
‐ Lateral, perpendicular to the crank arm and to the rotation plane.

It's possible to evaluate the effectiveness of the applied force over the crank arm rotating cycle through the effectiveness index *r*, a dimensionless ratio described by Equation (1):

(1) $$r=\frac{\text{Effective force}(\text{perpendicular})\left[N\right]}{\text{Total applied force}[N]}$$

This index can take values between −1 (when all the force is applied in the perpendicular direction, in each way that it is against the rotation of the crank arm—least effective) and +1 (all the force is applied in the perpendicular direction, and contributes with the crank arm rotation—most effective). Studies [9] highlight that, as applied force is a product of muscular action, a low effectiveness index is understood as inefficient muscular work and, therefore, a waste of energy.

By measuring the forces in both crank arms, it is possible to analyze and compare the contribution of each leg. The bilateral pedaling asymmetry is the subject of several studies, especially as it may negatively influence an athlete's performance and even cause injuries [10]. As seen in [11,12], the typical asymmetry for elite athletes varies in the range of 5% to 38% reaching up to 60% in some cases. The dominant leg identification can help to develop individualized training programs.

Nearly all of the studies about force orientation and pedaling techniques are conducted in laboratories, with the aid of training rollers or cycle ergometers. Although they have a great value for the dynamic's comprehension of the processes, tests in laboratory do not reflect the real competition conditions, including slope and even motivation difference. Through the development of wireless transmission systems or even telemetry systems, it becomes possible to build a system to measure the applied force components and other variables of interest directly in the field during competitions.

While pedaling, the cyclist's goal is to transmit a torque to the gear systems formed by the bicycle chain and gears. Once the crank arm length is known, it can be combined with the effective force applied in this crank arm to find the applied torque as shown in Equation (2):

(2) $$T=F_{\text{effective}}\times L$$

in which *T* [N·m] is the applied torque, F*_effective_* [N] is the effective component of the applied force and *L* [m] is the length of the crank arm. With the applied torque information, the mechanical power is given by Equation (3):

(3) P=T×ω

in which *P* [W] is the applied power, *T* [N·m] is the applied Torque and *ω* [rad/s] is the crank arm angular velocity. In cycling, the crank arm angular velocity is most commonly measured in RPM, and it is called cadence. Equation (3) can be coverted to Equation (4) to obtain the instantaneous applied power through the cadence:

(4) $$P=\frac{T\times 2\pi \times C}{60}$$

in which *P* [W] is the applied power, *T* [N·m] is the applied torque, and *C* [RPM] is the cadence. The mechanical power produced by the athlete is one of the basic parameters in most performed ergometric tests. In the science of cycling, the average power importance as a performance parameter as proven by [12], enabling the analysis of the athlete's performance in several manners.

## 3. Methodology and Hardware Description

**Figure 2.**
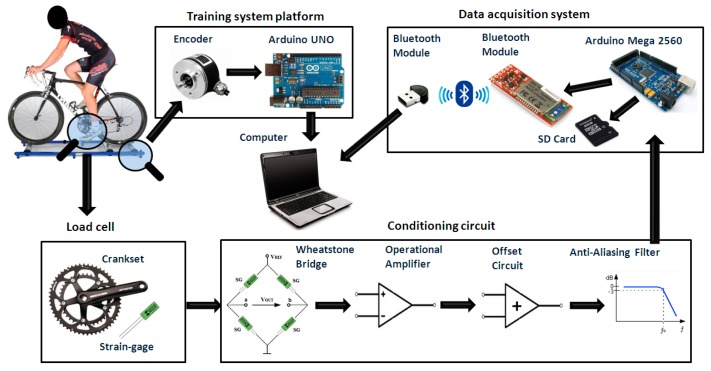
System block diagram.

Figure 2 shows the system block diagram proposed in this paper. The application of force on the crankset deforms the load cell and causes an imbalance in the Wheatstone bridge, followed by an amplification, filtering and normalization in the signal conditioner circuit [13]. The circuit's outputs are connected in a system that writes the data to a SD card and sends results via wireless communication to a computer using MATLAB, where the data is acquired and analyzed. Furthermore, an encoder installed at the training rollers allows the acquisition of the bike's wheel speed. Thus, the speed and the force applied by the cyclist are analyzed at the same time.

### 3.1. Load Cell Design

**Figure 3.**
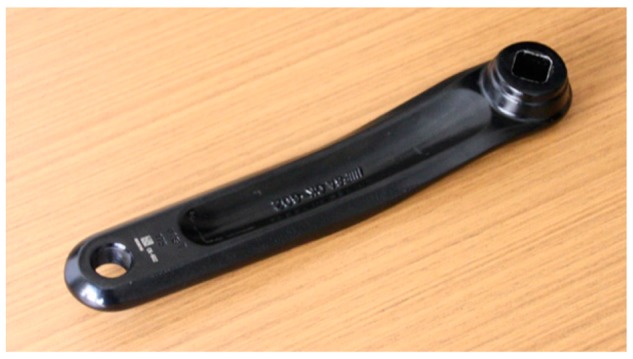
Commercial crank arm photo.

For the load cell's mechanical structure implementation, a commercial Full Speed Ahead (FSA) FSA CK-602 175 mm model crankset (VERO, Cyrus Way, WA, USA), in AL6061 aluminum alloy (Figure 3) was chosen. The choice is justified by the use of this crankset by many cyclists, both professionals and amateurs, in competitions, and its usage in several road bikes and commercial pedals. Moreover, its structure and its mechanical resistance are guaranteed by the manufacturer.

A mechanical stress analysis checks whether the result is within the limit of the material and determines the mechanical limits of the structure [13,14]. Moreover, one should evaluate the resonant dynamic behavior of the structure to determine whether it can have significant influence on the output variables of the load cell [13]. The virtual crankset was built using the Loft tool provided by SOLIDWORKS Simulation. Altogether, 14 profiles were created along the longitudinal axis of the piece (from 0 up to 110 mm) and were interconnected by guide curves through the Spline tool. This same procedure was used to build both ends.

**Figure 4.**
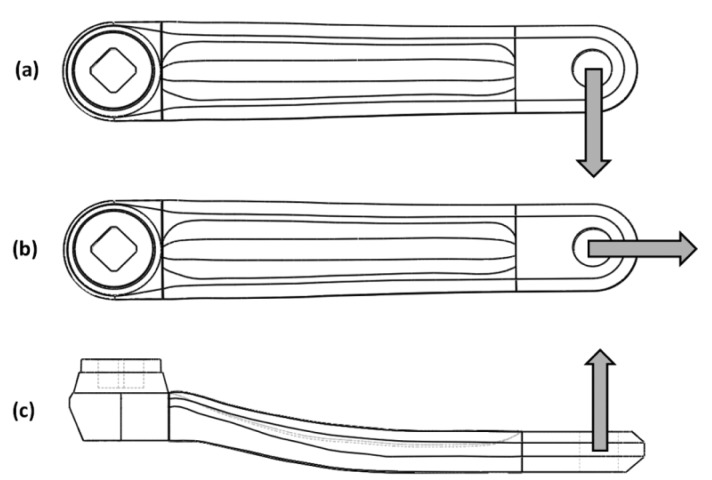
Direction of the applied force component in the crankset: (**a**) perpendicular force; (**b**) parallel force; (**c**) lateral force.

Initially it were performed static simulations of the previously generated model to analyze the levels of mechanical stress and deformation of the structure for a given load representing the cyclist's applied force. Considering that the total applied force to each pedal hardly exceeds the total rider weight [10], it was stipulated a maximum loading of 800 N, which corresponds to the limiting case of an athlete whose mass is approximately 80 kg. Thus, the model was fixed, simulating the central axis where the crankset is attached, and loads were applied in parallel, lateral and perpendicular directions to the longitudinal axis. Figure 4 represents these directions in the positive axes. This convention will be adopted throughout this paper. This test result is presented in Section 4.

Then, dynamics simulations were performed to observe the structure resonance modes. These modes determine the resonant frequencies of oscillation that can damage or influence the results of the load cell. Thus, the operating range of the cell should be below the first mode. The simulations indicated the first five structure resonance modes, which are found in Section 4.

In order to compare the dynamic simulations results, an experimental dynamic test was carried out. The crankset was attached to a testing structure in a similar way as in the bicycle and a DeltaTron 4520 (Bruel & Kjaer, Skodsborgvej, Denmark) triaxial accelerometer was placed on the crankset near where the pedal was attached. This experiment consisted of striking the crankset in the effective component's direction with a hammer, getting signals from the accelerometer. The data acquisition was performed by a specific accelerometer measurement system, connected to a personal computer, composed by NI SCXI 1530 and NI SCXI 1600 modules (National Instruments, Austin, TX, USA). The data was acquired using LabVIEW and analyzed through MATLAB for the frequency response. These experimental results can be found in Section 4.

### 3.2. Signal Conditioner

**Figure 5.**
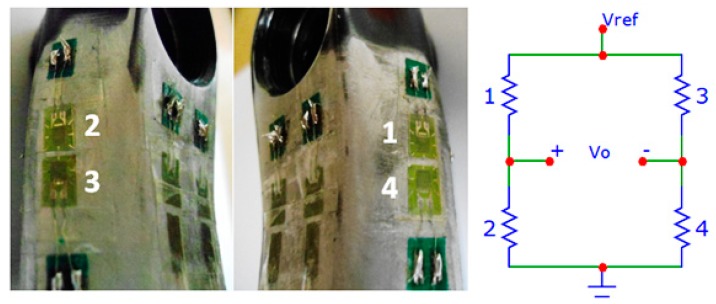
350.6 Ω strain gauges and bridge connections.

Two types of strain gauges were used (with aluminum temperature compensation) due to the size of the region of bonding, their models are: KFG-2-350-C1-23 (350.6 ± 0.6 Ω and Gauge factor 2.11% ± 1.0%); and KFG-2-120-C1-23 (120.2 ± 0.2 Ω and Gauge factor 2.10% ± 1.0%) (KYOWA Electronic Instruments Co., Chofugaoka, Tokyo, Japan). Figure 5 shows in detail the strain gauges bonded on the crankset and their connections in the Wheatstone bridge for measuring loads in the perpendicular direction. To measure the deformation due perpendicular and lateral loads, 350.6 Ω and 120.2 Ω strain gauges were used, respectively, in full-bridge configuration. To measure the deformation due to parallel loads, two 350.6 Ω active strain gauges and two 350.6 Ω dummy strain gauges were used, because both sides of the piece suffer tension (or compression). These dummy sensors were bonded in a region where the deformation of the piece was negligible in relation to the principal strains.

As the rider applies most of the effort during cycling in the perpendicular direction, the conditioning circuit for this direction was designed for a range of −785 N up to 785 N (corresponding to a 80 kg mass loading). For the other two directions (parallel and lateral), according to data from the literature review, the conditioning circuit was designed for a range of −196 N up to 196 N (corresponding to a 20 kg loading). To facilitate understanding of the studies, it was decided to divide the conditioning circuit into six channels as follows:

‐ Channels 1, 2 and 3 represent, respectively, the perpendicular, parallel and lateral components of left crank arm (crank arm without chainring);
‐ Channels 4, 5 and 6 represent, respectively, the perpendicular, parallel and lateral components of right crank arm (crank arm with chainring).

A deformation test of the load cell was carried out to develop the conditioning circuit more accurately. For this experiment, the crankset was attached in a similar way to the bicycle and loads up to 62 kg were applied in perpendicular and parallel directions. In lateral direction, loads up to 37 kg were applied in order to avoid damaging the piece. For conditioning and data acquisition of this experiment, it was used a NI-9237 module (National Instruments), that provides the results in strain. This module contains its own internal power supply system of reference for the Wheatstone bridge. Thus, the bridge was built connecting the strain gauges to this system through a RJ50 cable and the data was acquired through LabVIEW.

**Figure 6.**
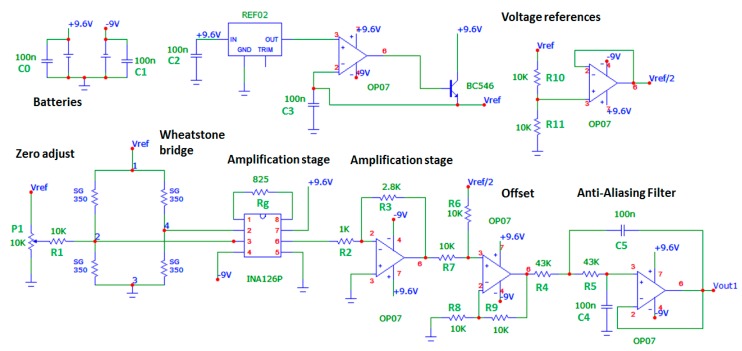
Channel 1's conditioning circuit.

**Figure 7.**
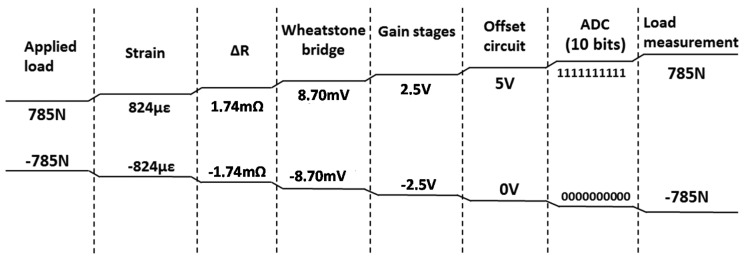
Channel 1's proposed measurement chain.

Two batteries, 9 V and 400 mAh, respectively, were connected in parallel to provide a negative voltage, and a set of eight AA batteries, 1.2 V and 2300 mAh, respectively, connected in series, totaling 9.6 V, for the conditioning circuit's positive supply voltage. The stability of the power supply of the Wheatstone bridge channels 1, 2, 4 and 5 is guaranteed by the reference voltage integrated circuit REF02 with 5.0 V ± 0.3% accuracy. To supply channels 3 and 6, the reference voltage integrated circuit REF03 with 2.5 V ± 0.6% accuracy is used. The first gain stage is performed by INA126 instrumentation amplifier and the active elements of all other stages are OP07 operational amplifiers. Next, a Vref/2 offset is added to the signal in order to normalize it to the analog-to-digital converter input, set to Vref. Finally, a second-order filter, whose cutoff frequency is 24 Hz, removes the high-frequency spectral components. The channel 1's voltage output is described by Equation (5). Channel 1's conditioning circuit electric diagram is shown in Figures 6 and 7 show the channel 1's proposed measure chain, as an example. The system is designed in a way that the efforts range corresponds to the maximum excursion of the analog-to-digital converter of 10-bit from Arduino board:

(5) $$V_{\text{out}1}=G_{1}.G_{2}.\left(\frac{R_{6}}{R_{6}+R_{7}}\right).\left(\frac{R_{9}}{R_{8}}+1\right)\cdot V_{o₋\text{brigde}1}+\left(\frac{R_{7}}{R_{6}+R_{7}}\right).\left(\frac{R_{11}}{R_{10}+R_{11}}\right).\left(\frac{R_{9}}{R_{8}}+1\right)\cdot V_{\text{ref}}$$

in which *V_out_*_1_ [V] is the channel 1's voltage output. *G*_1_ and *G*_2_ are the gains of the first and second gain stages, respectively. *R*_6_, *R*_7_, *R*_8_ and *R*_9_ [Ω] are the electrical resistances of the offset stage. *V_o_bridge_*_1_ [V] is the Wheatstone bridge's output voltage. *R*_10_ and *R*_11_ [Ω] are the electrical resistances of the voltage reference circuit. *V_ref_* [V] is the output voltage of the voltage reference circuit.

The static calibration was performed in order to obtain the experimental transfer function for each channel conditioning load cell. Thus, the crankset was attached in the same way as the other tests and loads up to 60 kg were applied in perpendicular and parallel directions and up to 20 kg in lateral direction. The conditioning circuit for this experiment was powered by a POL-16E symmetric model voltage source (Politerm Instrumentos de Medição Ltda, São Paulo, São Paulo, Brazil). The digital precision multimeter with a 6½ digit resolution DMM 4050 model (Tektronix, Inc., Beaverton, OR, USA) was used to measure the voltage output of each channel. The multimeter was set to get the arithmetic mean of 30 successive measurements of voltage to have a greater reliability in measurement.

### 3.3. Data Acquisition System and Training System Platform

One of the essential characteristics of the proposed system is allowing the transmission and storage of the data generated by the conditioning circuit. To accomplish this task, three devices were used: a board prototyping Arduino MEGA 2560, an OpenLog datalogger module, and a BlueSMIRF Gold Bluetooth module [15].

The system power is shared with the conditioning circuit so that the Arduino board is powered with 9.6 V. The 6 channels of conditioning are connected to the analog inputs of the Arduino board, and then digitized by the analog-digital converter (ADC) of the microcontroller through the *analogRead()* function already present in the function library of the Arduino IDE. The ADC of microcontroller Arduino board has 10 bit resolution and input from 0 V to 5 V, therefore, generating a value from 0 up to 1023. In order to reduce fluctuations in the ADC measurement, the 5 V output voltage reference provided by REF02 was used as a reference voltage for the conversion.

The Gold BlueSMIRF Bluetooth module is powered by the Arduino board's 5 V regulated output voltage and provides a wireless serial communication able to work at any baud rate between 2400 and 115,200 bps operating in the frequency band between 2.4 and 2.524 GHz. The TX and RX pins of the module are connected to the respective RX1 (pin 19) and TX1 (pin 18) pins of Arduino board's serial port number 1. Once the connection between the Bluetooth module and the Arduino was established, it was possible to configure the speed of this connection to 115,200 bps [15].

**Figure 8.**
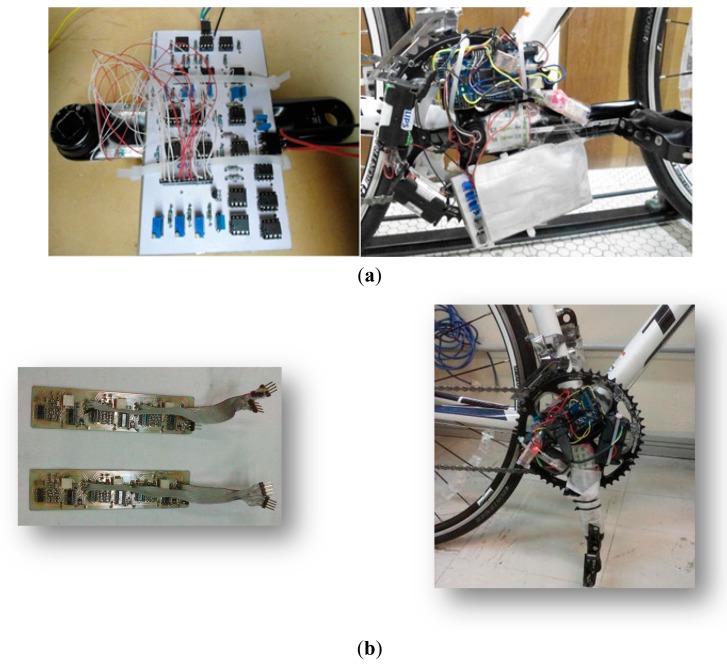
Force platform assembled: (**a**) first prototype and (**b**) final prototype (111.13 × 26.67 mm).

**Figure 9.**
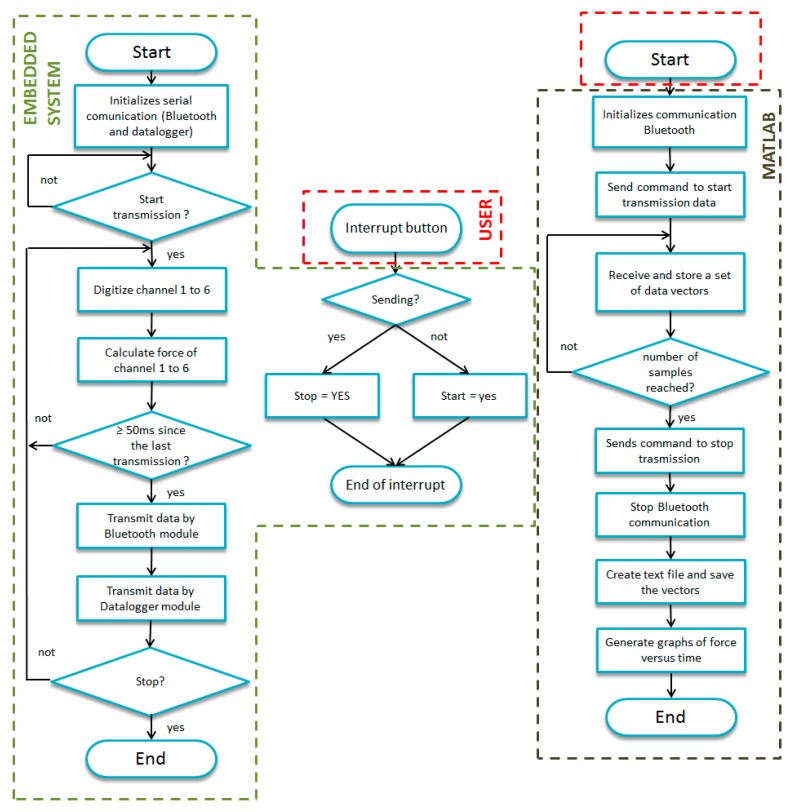
Flowchart of the routine implemented in the Arduino board's microcontroller and MATLAB.

The module is powered by the output Arduino board's 3.3 V voltage regulated pin, and communicates via serial at a rate that can be chosen between 2400 and 115,200. A rate of 9600 bps was chosen for the data recording on a micro SD card with a 4 GB FAT16 formatting type. This module has its TX and RX pins connected to RX2 (pin 17) and TX2 (pin 16) pins of the Arduino board's serial port number 2 and each time the circuit is switched on, it creates a new sequential “LOG0000x.TXT” text file type. Figure 8 shows the force platform assembled and Figure 9 shows the flowchart of the routine implemented in the Arduino board's microcontroller and on MATLAB.

The training platform system is composed of: three rolls of the same outer diameter, two of which are located under the rear wheel of the bicycle, and the third is located under the front wheel. The front roller is driven via a belt pulled by one of the rear rollers, which are pulled directly from the rear wheel of the bicycle; a system for measuring the speed of rotation of the rollers comprises an incremental optical encoder (model 2R2000-DS-CON—Scancon Encoders A/S, Tranevang, Lillerød, Danmark) coupled to one of the rollers [16]; and a data acquisition system composed of a board prototyping Arduino UNO.

The force components measurement tests were performed with a road bike (Trek 1.5 model) and an amateur cyclist, weighing approximately 75 kg, in a laboratory environment (using the training platform system developed) and on-track. The test results are in Section 4.

## 4. Experimental Section

### 4.1. Structural Analysis

**Figure 10.**
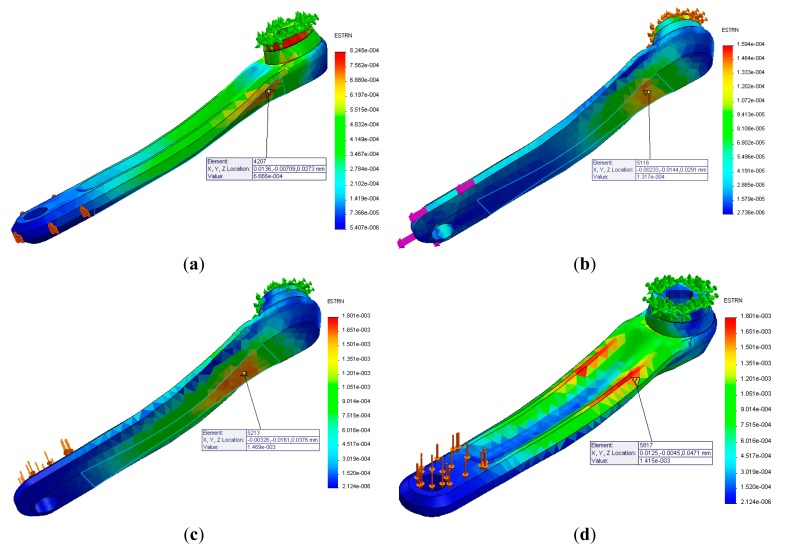
Static simulations results for 800N (**a**) perpendicular; (**b**) parallel; (**c**) lateral; (**d**) lateral loading.

Figure 10 shows the static simulated results for the 800 N loading directions condition. For the evaluated cases, the main strain points have values around 666 με, 131 με and 1415 με for perpendicular, parallel and lateral loads, respectively; which identified these regions as ideal for the strain gauges location. The structural integrity analysis reveals the most critical condition, in which a tensile force could reach 197 MPa being this value well below the material's resistance to fatigue, which is 275 MPa. However, it is noted that this structure safety factor is only 1.4.

**Figure 11.**
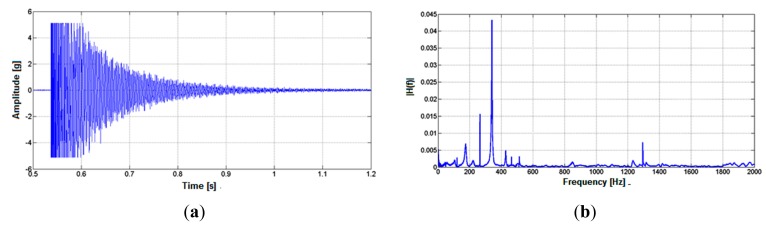
Vibration test: dynamic behavior in the (**a**) time and (**b**) frequency domain.

From Figure 10b,c, it is noted that there is a single main region of deformation when a load is applied in the parallel and lateral direction. This result means that it will not be possible to isolate these components of force and the load cell will measure the combination of both components. Furthermore, it is noted that the deformation due to the lateral loading direction is approximately 12 times greater than that due the parallel loading direction. The first fundamental vibration mode, was found to be 350 Hz. It should be noted that the experimental test performed using the impact technique resulted in a fundamental natural frequency of 340 Hz as showed in Figure 11. So, the experimental results are in agreement with the simulation. It is important to note that this frequency is higher than the primary excitation frequency typically generated by an athlete (0.5 Hz to 2 Hz) [9].

### 4.2. Deformation Experiment

**Figure 12.**
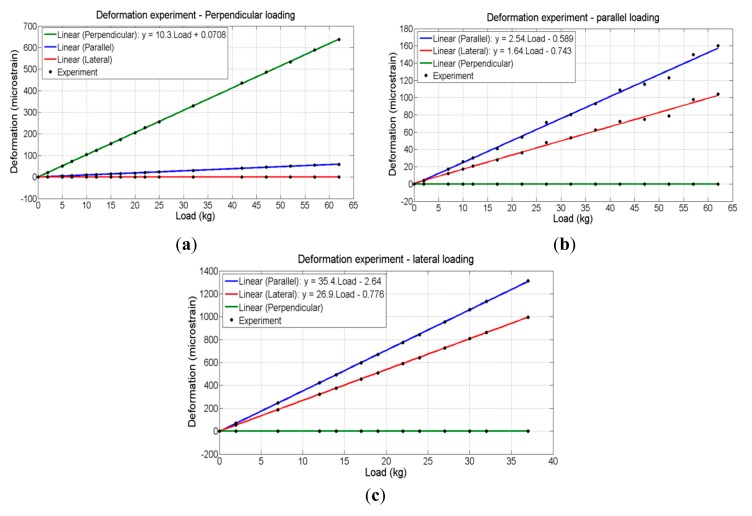
Deformation experiment results for the left load cell: (**a**) perpendicular; (**b**) parallel; (**c**) lateral loading.

The experimental transfer function for each direction of deformation of the left load cell (left crank) are shown in Figure 12 ((a) perpendicular load, (b) parallel load and (c) lateral load).

In Figure 12a it is observed that most of the deformation occurs in the same direction of load application, in this case, the perpendicular direction. From Figure 12b,c, it is observed that, as seen in the static simulation, the deformation due to the application of load in lateral direction is greater than that due to load application in the parallel direction. Therefore, it was chosen to use the transfer functions for a load in the lateral direction for both designs of channels 3 and 6 (lateral force) and channels 2 and 5 (parallel force) to avoid saturation. Thus, it was possible to define the maximum loads and maximum and minimum deformations corresponding to the design of each channel conditioning as shown in Table 1. It should be noted from this same table that the maximum linearity error obtained in the experiment was approximately 1%. The results obtained for the right load cell were similar.

### 4.3. Static Calibration Experiment and Conditioning Circuit Validation

**Figure 13.**
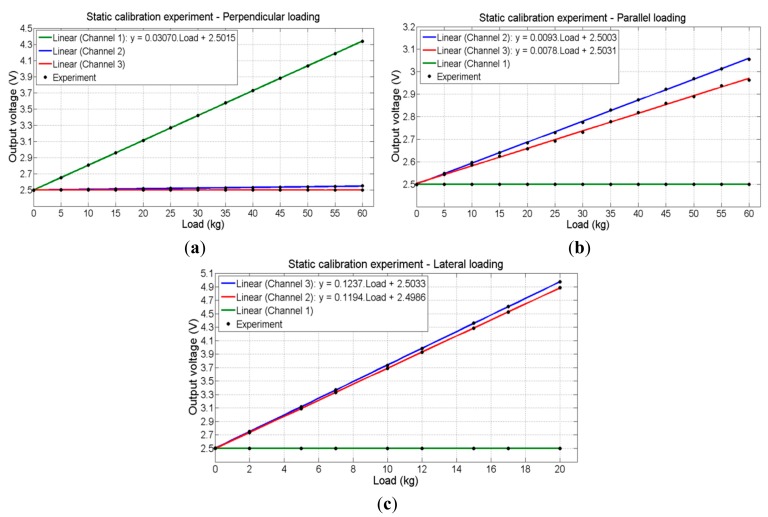
Deformation experiment: (**a**) perpendicular; (**b**) parallel; (**c**) lateral loading.

Figure 13a–c shows the curve of the output voltage as a function of load application in perpendicular, parallel and lateral directions, respectively, (*i.e.*, channels 1, 2 and 3). The experimental transfer function obtained for each conditioning channel is also displayed in these figures. The experimental transfer function defined for each channel as well as the sensitivity and the linearity error of these channels are shown in Table 2.

Due to limitations in the calibration test, the experimental transfer function of channel 1 (and channel 4) was obtained at a maximum load of 60 kg, as shown in Figure 13a. Thus, according to these transfer functions, at a maximum load defined as 80 kg, the voltage output of channel 1 is 4.96 V (and channel 4's is 4.90 V). From Figure 13b,c, it is observed, as expected, that for the load in the lateral direction, both channels 2 and 3 (and both channels 5 and 6) have a similar behavior. At the defined maximum load of 20 kg, the voltage output of these channels is about 5 V. The obtained results for the right load cell are similar.

### 4.4. Tests with the Complete System

**Figure 14.**
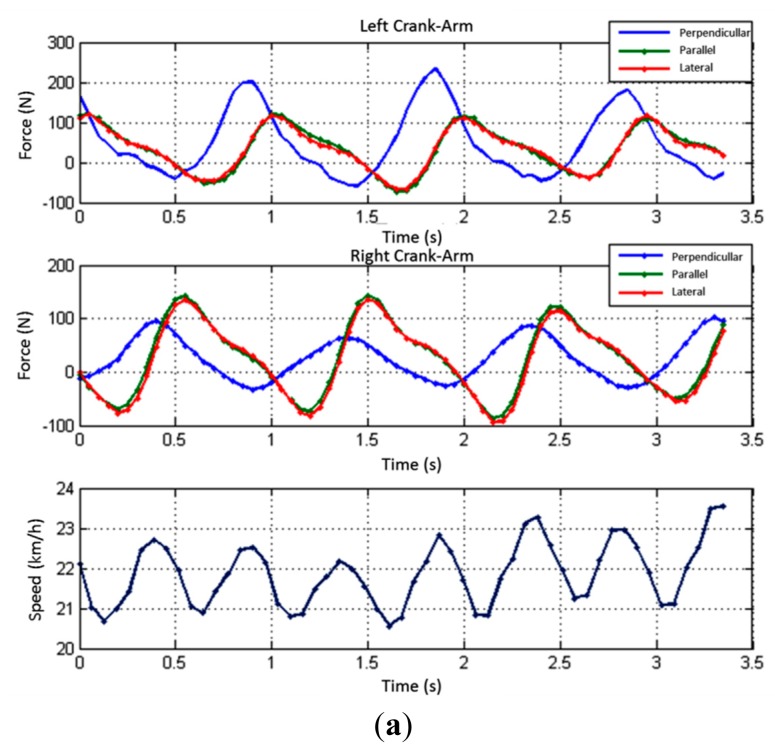

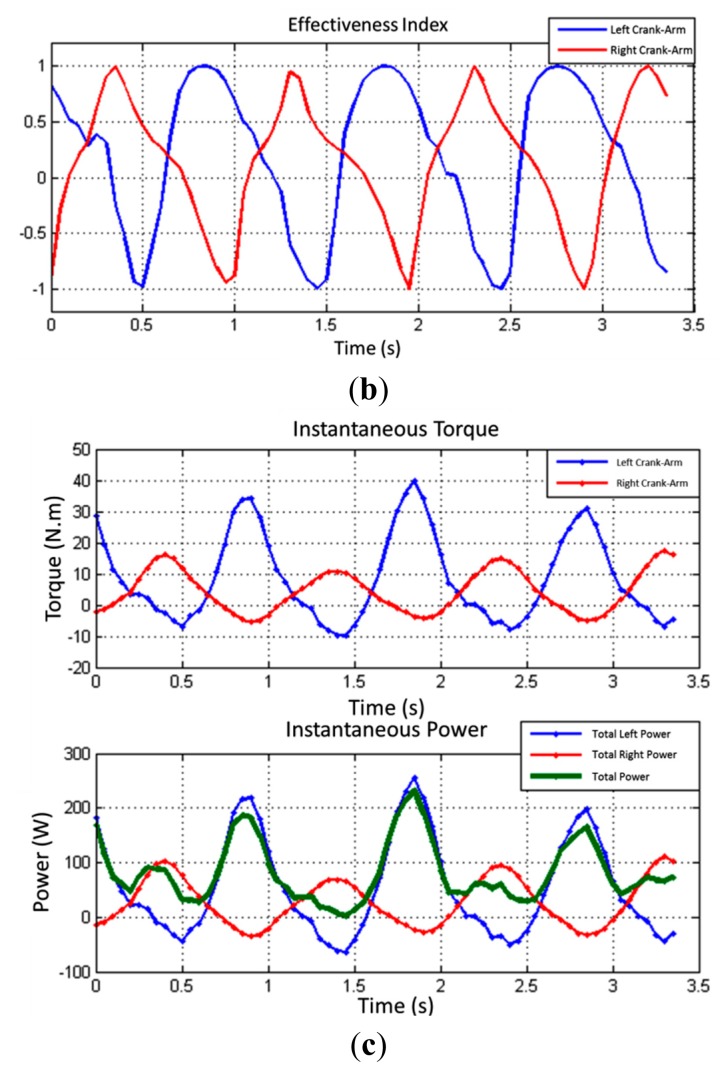
Tests in laboratory: (**a**) Forces and Speed; (**b**) Effectiveness Index; (**c**) Torque and Power.

In the first test conducted using the instrumented training roller, the amateur cyclist tried to maintain a constant speed with the aid of a commercial speedometer. Data from the conditioning circuit of the force platform was acquired for 40 s via Bluetooth. Simultaneously, information of the speed of the rollers was obtained via serial communication through the Arduino UNO module. Figure 14a shows a range of 3.35 s withdrawn from this test.

Figure 14a shows the contribution of each cycle of pedaling for each crank arm on the specific speed. The average speed in the observed time interval is 21.85 km/h. It is possible to note a cyclic behavior of the force components along the pedaling as previously shown by [17–23].

The effectiveness index of the forces applied to each crank arm was calculated according to Equation (1). Figure 14b shows the instantaneous effectiveness index in the observed time interval for each crank arm. It is noticed that, as the peaks of perpendicular forces, the effectiveness index of the right and left crank arms intercalate in time due to the physical coupling of the crank arms to the crank spindle in opposite directions. In the observed time interval, the average effectiveness indexes obtained were 17.6% to the left leg and 12.7% to the right leg. The average asymmetry obtained was 61.6%, being the minimum and maximum observed asymmetries respectively 51.6% and 73.1%. From this information, a coach can develop strategies for specific training to reduce this difference between sides and avoid possible injuries.

Although the system does not have a crank arm angle meter, it is possible to estimate the cadence by considering that the peak effective force observed in a crank arm revolution always happens in the same angular position. This approximation is justified because the force applied by the cyclist is greater when it is applied at an angle of 90° with the crank arm. Therefore, the observed average cadence is 61.05 RPM.

Having the cadence in the interval, and calculating the instantaneous torque at each point, the instantaneous power applied to each crank arm is obtained, as shown in Figure 14c. The observed average power applied by the left leg is 55.6 W, and by the right, 25.8 W. The total average power that is transferred to the crown/chain set is 81.4 W. The asymmetry between the average power applied by the left and right legs in the observed interval is 53.6%.

**Figure 15.**
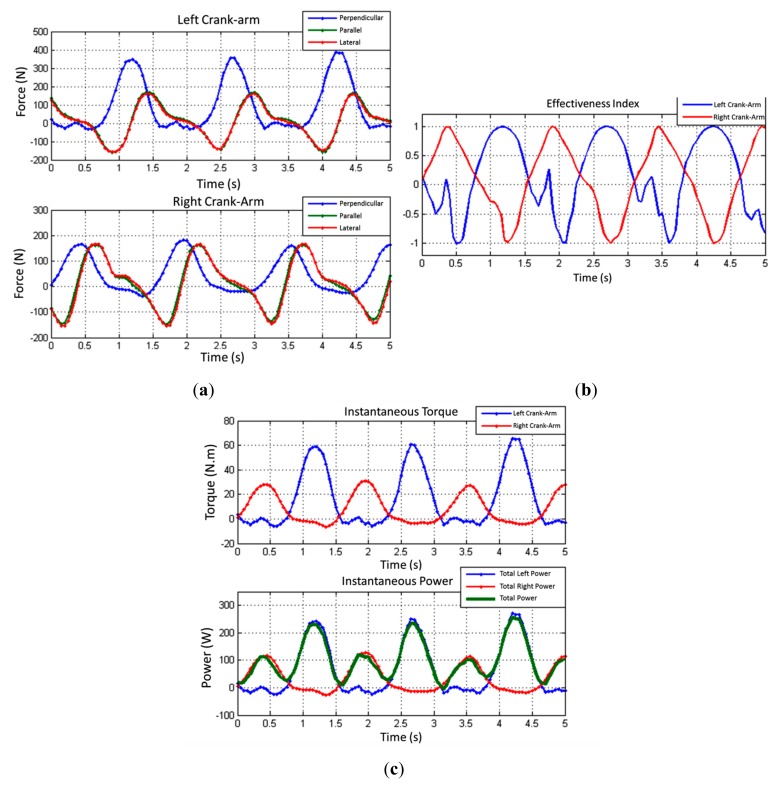
Outdoor test: (**a**) Forces; (**b**) Effectiveness Index; (**c**) Torque and Power.

As a second test, the same cyclist rode about 3 min in an external environment, on a closed circuit road track. The data was stored by the datalogger system in a SD card. Once the test was finished, the data from the SD card was analyzed on a computer using a MATLAB routine. Figure 15a shows a 5 s excerpt of the outdoor test.

It was observed an average effectiveness index of 19.1% for the left leg, and 6.72% to the right leg. Like the test conducted on the training rollers, the asymmetry between the maximum forces applied at each crank arm was calculated. An average asymmetry of 51.8% was observed. The average cadence observed was 39.34 RPM. Compared with the laboratory test, it is noted that the cadence is lower, mainly due to the higher opposition to the movement of the bike.

The average power applied effectively by the left leg in the observed interval was 64.2 W and 34.0 W for the right leg. The total average power in the interval, transferred to the crown, was 98.2 W. It is noticeable that even if the maximum torque peaks doubled compared to the test conducted in laboratory, the total average power applied by the cyclist in the perpendicular direction increased only 20%. This is due to the application of torque at a lower cadence, compared to the test on the training rollers. Also in this test, it was found that the asymmetry between the average power applied by each leg for the analyzed interval was 47%.

## 5. Conclusions

Through the initial bibliographic review realized in this research [24], it was possible to have a global perspective of the biomechanics studies applied to cycling [1–12,17–23,25–27], revealing new challenges. A static simulation of the crank arm under load showed the main deformation points, whereas the structure dynamic response showed a primary vibration frequency of 340.3 Hz, is above the higher crank arm loading frequency—the cadence of pedaling, with a practical maximum of 2 Hz. The complete load cell system showed a nonlinearity error of less than 1%, with the system output excursioning through all the analogic-to-digital converter input range.

The developed training rollers, with the aid of an encoder coupled to one of the rollers, were used to measure the bicycle's wheel speed during laboratory tests. As a differential regarding other studies in this area, the instrumented rollers enable the integrated analysis of the applied forces and the bicycle speed. With the complete system, it was possible to measure the force components and analyzed its efficiency and asymmetry. The outdoor tests showed peaks of 386 N of effective force applied by the left leg, generating a medium power of 98.2 W during the analyzed period. An asymmetry of 47% was observed in relation to the power between legs.

From these results, it is concluded that the main goal of the research was achieved. The developed system is a functional wireless force platform capable of measuring the forces acting in the crank arm during real competition usage. Moreover, it may act as an experimental basis for the development of new equipment and new studies on the biomechanics of cycling.

## Figures and Tables

**Table 1.** Load cell characteristics for conditioning channels design.

**Channel**	**Transfer Function**	**Linearity Error (%)**	**Load (kg)**	**Load (N)**	**Deformation (με)**
1	*y* = 10.3 × *Load* + 0.0708	0.36	±80	±785	±824
2	*y* = 35.4 × *Load* − 2.64	0.28	±20	±196	±705
3	*y* = 26.9 × *Load* − 0.776	0.29	±20	±196	±537
4	*y* = 8.65 × *Load* + 0.713	0.61	±80	±785	±693
5	*y* = 27.7 × *Load* − 2.64	0.26	±20	±196	±551
6	*y* = 27.0 × *Load* − 7.43	1.1	±20	±196	±533

**Table 2.** Conditioning channels characteristics.

**Channel**	**Transfer Function**	**Linearity Error (%)**	**Sensibility (V/kg)**	**Sensibility (mV/N)**
1	*S*_1_ = 0.0307 × *Load* + 2.5015	0.09	0.0307	3.131
2	*S*_2_ = 0.1194 × *Load* + 2.4986	0.12	0.1194	12.17
3	*S*_3_ = 0.1237 × *Load* + 2.5033	0.13	0.1237	12.61
4	*S*_4_ = 0.0296 × *Load* + 2.5144	0.22	0.0296	3.018
5	*S*_5_ = 0.1214 × *Load* + 2.4964	0.13	0.1214	12.38
6	*S*_6_ = 0.1189 × *Load* + 2.4935	0.14	0.1189	12.12
